# Supplementary figures and images for: The Role of Soluble Adenylyl Cyclase in the Regulation of Flagellar Motility in Ascidian Sperm
Source: Biomolecules. 2023 Oct 30;13(11):1594. doi: 10.3390/biom13111594 (PMC10668965; doi:10.3390/biom13111594)

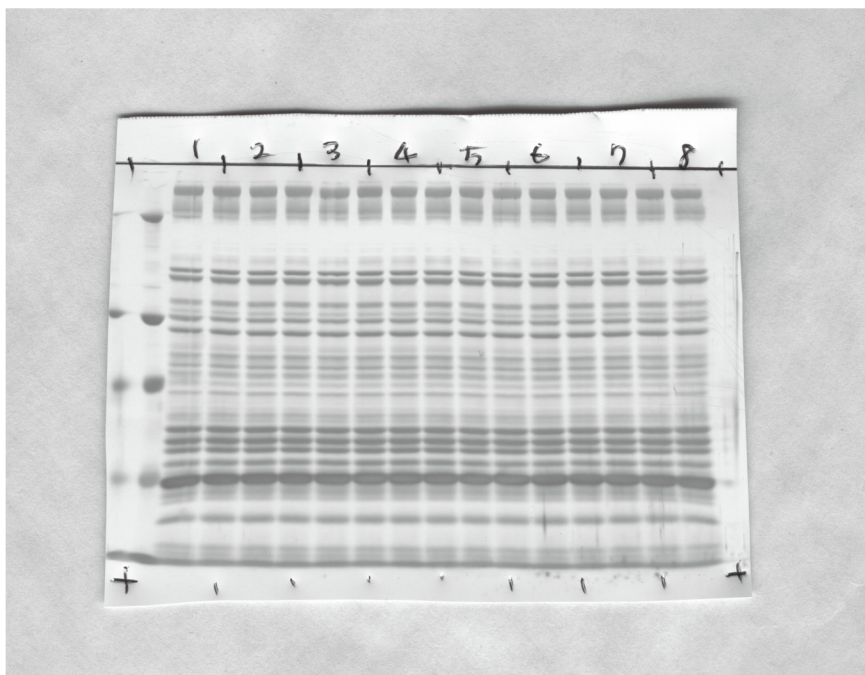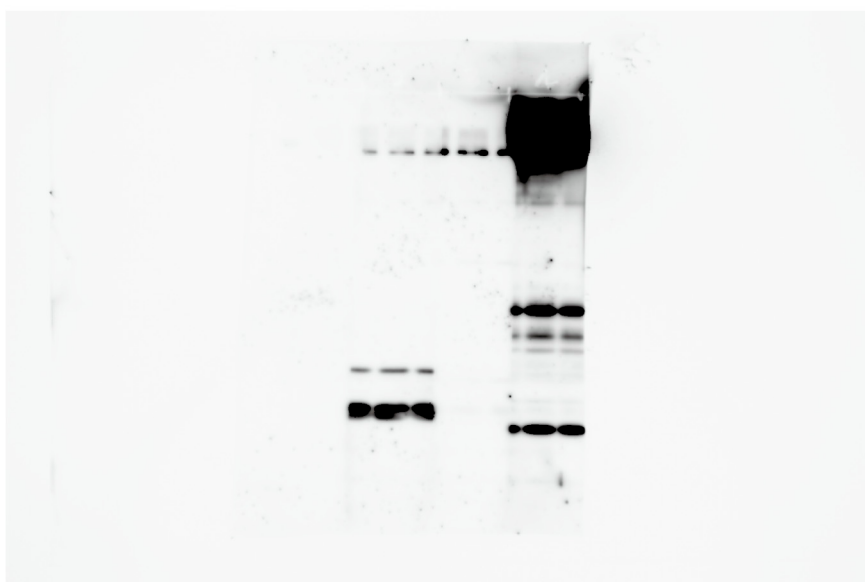

Figure S1: Original images of Figure 1B.

Supplement: Supplementary file 1 [file biomolecules-13-01594-s001.zip › biomolecules-2581731-supplementary.pdf]
